# Chromosome-specific retention of cancer-associated DNA hypermethylation following pharmacological inhibition of DNMT1

**DOI:** 10.1038/s42003-022-03509-3

**Published:** 2022-06-02

**Authors:** Ashley K. Wiseman, Rochelle L. Tiedemann, Huihui Fan, Hui Shen, Zachary Madaj, Michael T. McCabe, Melissa B. Pappalardi, Peter A. Jones

**Affiliations:** 1 Department of Epigenetics, Van Andel Institute, Grand Rapids, MI 49503 USA; 2 Center for Precision Health, School of Biomedical Informatics, The University of Texas Health Science Center at Houston, Houston, TX 77030 USA; 3 Cancer Epigenetics Research Unit, Oncology R&D, GlaxoSmithKline, Collegeville, PA USA

**Keywords:** DNA methylation, Cancer epigenetics

## Abstract

The DNA methylation status of the X-chromosome in cancer cells is often overlooked because of computational difficulties. Most of the CpG islands on the X-chromosome are mono-allelically methylated in normal female cells and only present as a single copy in male cells. We treated two colorectal cancer cell lines from a male (HCT116) and a female (RKO) with increasing doses of a DNA methyltransferase 1 (DNMT1)-specific inhibitor (GSK3685032/GSK5032) over several months to remove as much non-essential CpG methylation as possible. Profiling of the remaining DNA methylome revealed an unexpected, enriched retention of DNA methylation on the X-chromosome. Strikingly, the identified retained X-chromosome DNA methylation patterns accurately predicted *de novo* DNA hypermethylation in colon cancer patient methylomes in the TCGA COAD/READ cohort. These results suggest that a re-examination of tumors for X-linked DNA methylation changes may enable greater understanding of the importance of epigenetic silencing of cancer related genes.

## Abstract (web summary)

Chromosome specific retention of cancer associated DNA hypermethylation following pharmacological inhibition of DNMT1 is shown, with residual CpG methylation on the X chromosome compared to autosomes, suggesting a separate mechanism of methylation.

## Introduction

The methylation of CpG dinucleotides has been known to be essential for mammalian development for many years^1^. In addition, alterations in methylation patterns, particularly the *de novo* methylation of CpG islands, are a feature of almost all human cancers^2,3^. While some proportion of the methylation in both normal and cancer cells is necessary for cellular viability, much of it seems to be dispensable and plays no known function. Cells in culture can exist and divide with drastically reduced methylation levels^4^. Distinguishing which sites require methylation for cell survival is difficult without genetic or pharmacological interventions which have limitations in the clarity of the data they provide. We previously used colon cancer cell lines obtained from the Vogelstein and Baylin laboratories^5,6^, which have genetic knockdowns and/or knockouts of two of the three known DNA methyltransferases (DNMTs), DNMT1 and DNMT3B, to discover driver methylation sites for viability^7^. These experiments identified several key genes which required silencing by promoter methylation for survival, in that their re-expression was associated with cell death.

The three known DNMTs, which all show preference for CpG sites, are expressed as multiple isoforms and seem to have different but overlapping functions in the cell^8,9^. DNMT1 performs a vital role in copying methylation patterns by converting hemi-methylated sites to full methylation following DNA replication, and also shows *de novo* activity^10^. DNMT3A1 and 3A2 are considered to be *de novo* enzymes in that they can apply methyl groups to either unmethylated or hemimethylated CpGs and do not have known preferences for particular genomic locations. DNMT3B on the other hand, is expressed in multiple catalytically active and inactive splice variants and is required for efficient methylation of gene bodies^11–13^. The catalytically inactive isoform (DNMT3B3) can also act as an accessory protein to bind to the nucleosomal acidic patch and anchor DNMT3A2 to nucleosomes^14^. Deciphering the relative contributions of these enzymatic functions to the structure and function of the epigenome has been difficult and has not been successful at a global level.

In the current experiments, we used a DNMT inhibitor, GSK3685032 (abbreviated GSK5032) which is specific for the so-called maintenance enzyme DNMT1, to obtain cells which were resistant to continuous exposure to increasing concentrations of the compound. The residual patterns of methylation, which presumably are mostly due to the activities of DNMT3A and DNMT3B isoforms, showed an unexpected dependency on chromosome location. In particular, promoter probes on the active X chromosome which had become *de novo* methylated in the cancer cells were preferentially resistant to DNMT1 inhibition compared to other chromosomes. Surprisingly, several of these probes were associated with genes strongly expressed in normal colonic tissue yet were frequently methylated in colorectal and other tumors in both males and females. The differential retention of DNA methylation on different chromosomes suggests that DNMT3A/3B isoforms may have chromosome-specific functions. Additionally, we have uncovered several new, to the best of our knowledge, cancer related genes which have been overlooked because of their location on the X chromosome.

## Results

### DNMT1 inhibition or knockdown cause genome wide demethylation

**Fig. 1:**
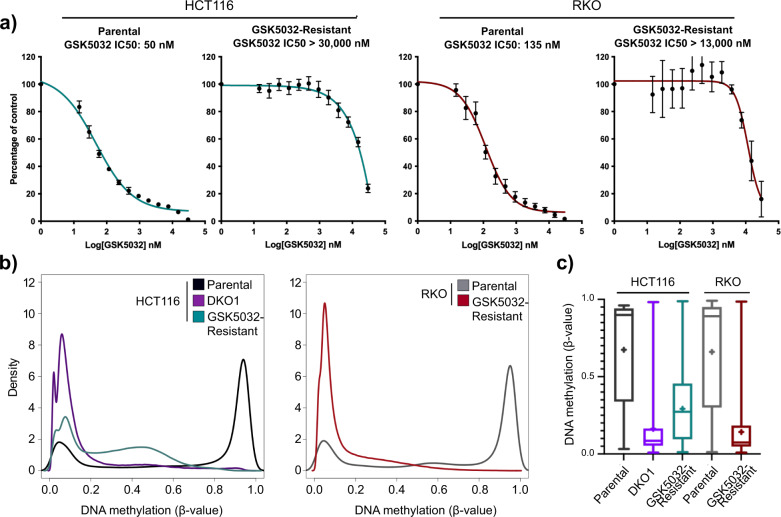
HCT116 and RKO GSK5032-resistant cells are globally demethylated. **a** Dose dependency curves showing that HCT116 and RKO cells long term treated with high doses of GSK5032 become resistant. Each point represents a percentage of surviving cells compared to cells treated with a vehicle control (*n* = 5 for HCT116 parental, *n* = 7 for HCT116 GSK5032-resistant, *n* = 3 for RKO parental, and *n* = 3 for RKO GSK5032-resistant). **b** DNA methylation density curves showing the distribution of the *β*-values on the Infinium EPIC methylation array (*n* = 627,029 probes). Probes with detection pval > 0.05 in any sample were removed from the analysis. *β*-values range from 0.0 to 1.0 where a *β*-value of 0.0 indicates completely unmethylated and a *β*-value of 1.0 indicates complete methylation. **c** Box plot of DNA methylation (*β*-value) distribution of all EPIC array probes for each individual sample queried in (**b**). Center line, median; box limits, upper and lower quartiles; whiskers, 5 and 95% percentile; ‘+’, mean; outliers removed for visualization purposes (*n* = 627,029 probes).

Our previous work demonstrated a global loss of DNA methylation with a very small proportion of promoter CpGs maintaining methylation in the absence of DNMT1 and DNMT3B^7^. Importantly, the DNA methylation that was maintained was associated with silencing of key genes whose repression is required for cancer cell survival in cell culture. However, our analysis of retained DNA methylation in this study was restricted to promoter CpGs due to the available technology at the time (Infinium 27k). Additionally, the two clones with concurrent knockdown of DNMT1 and knockout of DNMT3B used in the original study, HCT116 DKO8 and DKO1, demonstrated different degrees of DNA methylation loss and retention. In the present study, we aimed to expand on our initial work by profiling DNA methylation across the genome (Infinium MethylationEPIC array) and generating an additional stable cell model of severely impaired DNMT function in order to identify additional DNA methylation patterns that are essential for cancer cell survival. To generate such a model, we treated two colorectal cancer cell lines with increasing concentrations of GSK5032, a DNMT1 specific inhibitor, over several months. We first determined the IC50s for the HCT116 and RKO colorectal cancer cell lines before (parental lines) and after long term exposure (GSK5032-resistant lines) to increasing concentrations of the GSK5032 inhibitor (Fig. 1a). The parental cells, which were derived from a male and female respectively, showed IC50s of between 50 and 135 nM and both cell types became markedly resistant to the drug as shown by the IC50s increasing almost 1000-fold to over 30,000 nM for HCT116 and 13,000 nM for RKO. The doubling times of the treated (GSK5032-resistant) and HCT116 DKO1 cells, which have a hypomorphic version of the DNA methyltransferase 1 (*DNMT1*) and a complete knockout of *DNMT3B*^4,6^, were markedly increased (Supplementary Fig. 1a). The levels of RNA expression of several DNA methyltransferase isoforms showed minor changes as a result of drug treatment, with the exception of increased expression of *DNMT3A2* in treated or knockdown cells (Supplementary Fig. 1b). The data therefore show that the resistance is not due to a strong upregulation of DNMT1 or the other two methyltransferases.

Next, we used Infinium MethylationEPIC arrays to analyze the patterns of DNA methylation represented as beta values (*β*-value), which are defined as the ratio of methylated probe intensity to the overall intensity. HCT116 parental cells showed a strong bimodal distribution of *β*-values in which the majority of CpG probes queried are concentrated in either the unmethylated density peak (*β*-value $\cong$ 0.0) or the fully methylated density peak (*β*-value $\cong$ 1.0) (Fig. 1b, left). Conversely, HCT116 DKO1 and GSK5032-resistant cells showed no density of CpG probes that were fully methylated, but rather an enriched density of CpG probes that were unmethylated (Fig. 1b). Interestingly, the drug treated cells (HCT116 GSK5032-resistant) showed a shoulder of intermediate levels of methylation which was not present in the HCT116 DKO1 cells. Since the HCT116 GSK5032-resistant cells, unlike the HCT116 DKO1 cells, express DNMT3B it is possible that the shoulder is due to targeting of these CpGs by DNMT3B. The inhibitor also strongly reduced the methylation of highly methylated probes in RKO cells (Fig. 1b, right). The average *β*-values of all probes in both the HCT116 and RKO parental cell lines were reduced from about 0.66 to 0.29 and 0.14 respectively following long-term treatment (GSK5032-resistant) and 0.16 in HCT116 DKO1 cells (Fig. 1c).

### Probes on the X Chromosome are preferentially resistant to demethylation

**Fig. 2:**
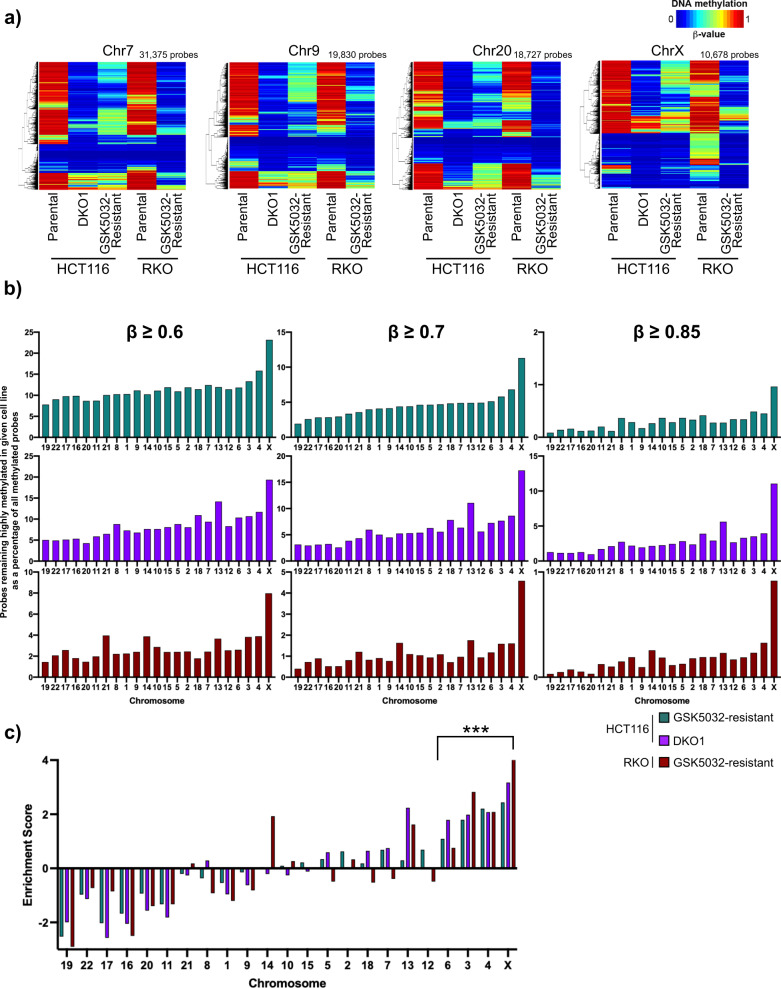
DNA methylation retention in DNMT compromised demethylated cell lines is concentrated on the X chromosome. **a** Heatmap of DNA methylation level (*β*-value) of all the CpG probes present on the indicated chromosome. *β*-values are represented by color scale where dark blue (colder colors) indicates no methylation and red (warmer colors) indicates complete methylation. Chr7 (*n* = 31,375 probes), 9 (*n* = 19,830 probes), 20 (*n* = 18,727 probes), and X (*n* = 10,678 probes) are a representative example of all chromosomes presented in Supplementary Fig. 2. Retained methylation is enriched on the X chromosome compared to the autosomal chromosomes as more probes have a high *β*-value following DNMT perturbation as indicated by the red color in these samples. **b** Percentage of CpG probes retaining high DNA methylation (*β*-value ≥ 0.6, 0.7, or 0.85) out of all highly methylated probes per chromosome after genetic knockdown in HCT116 DKO1 or treatment with GSK5032 in HCT116 and RKO (GSK5032-resistant). Chromosome order was ranked by least to most retained DNA methylation for HCT116 GSK5032-resistant cells with *β*-value ≥ 0.7. **c** Enrichment bias analysis of probes by chromosome retaining DNA methylation (*β*-value $\ge$ 0.7) in the indicated cell lines. Positive enrichment scores indicate more probes retained DNA methylation than expected while negative enrichment scores indicate fewer probes retained DNA methylation than expected by random chance. Enrichments were considered significant (***p value $\le$ 1.0 × 10^−6^) with hypergeometric testing.

Our next goal was to determine the extent of retained DNA methylation in our globally demethylated cancer cell culture models. First, we profiled the *β*-value of each individual CpG probe broken down by chromosome location for HCT116 (Parental, DKO1, and GSK5032-resistant) and RKO (Parental and GSK5032-resistant) cell line populations (Fig. 2a (Chr7,9,20,X); Supplementary Fig. 2 (all chromosomes)). The genetic knockdown (HCT116 DKO1) or pharmacological inhibition of DNMT1 (GSK5032-resistant) showed the extensive loss of DNA methylation seen in Fig. 1b, c, but a minority of CpG probes retained some DNA methylation (probes that demonstrate warmer heatmap colors) even after exhaustive drug treatment. Surprisingly, the X-chromosome showed more CpG probes that retained high levels of DNA methylation (as evident by increased warmer heatmap colors in the HCT116 DKO1 and both GSK5032-resistant lines) in comparison to the autosomal chromosomes (Fig. 2a and Supplementary Fig. 2). Similar but distinct patterns of CpG methylation in parental HCT116 or RKO cells were apparent.

To further dissect the distribution and location of retained DNA methylation in our DNMT disrupted cell lines (HCT116 DKO1, HCT116 and RKO GSK5032-resistant), we first used three different *β*-value cut-offs ranging from 0.6 to 0.85 to categorize highly methylated CpGs that retained DNA methylation (Fig. 2b). Next, we calculated the percentage of highly methylated CpG probes that retained DNA methylation on each chromosome in the cell lines with different *β*-value cutoffs for categorizing highly methylated probes. Finally, the data for each chromosome were ranked by increasing percentages in HCT116 GSK5032-resistant cells at a *β*-value of 0.7. Individual chromosomes differed markedly in their abilities to retain a high level of methylation. The patterns of retention were very similar under all conditions tested and showed a strong enrichment on the X chromosome in all demethylated cell lines (HCT116 DKO1, HCT116 GSK5032-resistant, and RKO GSK5032-resistant). Interestingly, the retention patterns on individual autosomes were similar in the three cell lines with chromosomes 19, 22, 17, and 16 being less likely to retain DNA methylation in all cases.

To ensure that the observed enrichment of retained DNA methylation on the X chromosome was not due to an inherent bias in the EPIC array composition, we analyzed our data in a number of ways. The probes on the array are distributed much as to be expected based on chromosome size increasing by their numerical designation (Supplementary Fig. 3a). The distribution of probes on the most demethylated chromosome (19) and the most resistant chromosome (X) were very similar with respect to their genomic annotations (Supplementary Fig. 3b). Therefore, the marked differences in retention of DNA methylation were not due to a skewed probe distribution. Also, these observations were not due to different basal levels of methylation of chromosomes in the parental cells, which showed a uniform percentage of probes methylated (*β*-value ≥ 0.7) in untreated cells, which was around 60% of probes for each chromosome (Supplementary Fig. 3c, d). Finally, we calculated the enrichment bias for DNA methylation retention on individual chromosomes (compared to the number of methylated probes (*β*-value $\ge$ 0.7) on each individual chromosome in the respective parental cell lines) and performed hypergeometric testing to determine the level of significance of the enrichment (Fig. 2c). While chromosomes 6, 3, and 4 also demonstrated significant enrichment of retained DNA methylation, the X chromosome consistently had the strongest enrichment score across all queried cell populations (HCT116 DKO1, GSK5032-resistant; RKO GSK5032-resistant) (Fig. 2c). Consistent with the lack of DNA methylation retention observed in our previous analysis (Fig. 2b), chromosomes 19, 22, 17, and 16 demonstrated negative enrichment scores indicating that they are selected against for retention of DNA methylation (Fig. 2c). Indeed, the fact that the same results were obtained in two cell lines treated with the DNMT1 inhibitor (HCT116 and RKO GSK5032-resistant) and in one cell line with a partial *DNMT1* knockout (HCT116 DKO1) suggests that particular methyltransferases may differentially act on the various chromosomes.

### CpGs that retain DNA methylation on the X-chromosome in the HCT116 GSK5032-resistant cells are either unmethylated or methylated in non-cancer human tissues

**Fig. 3:**
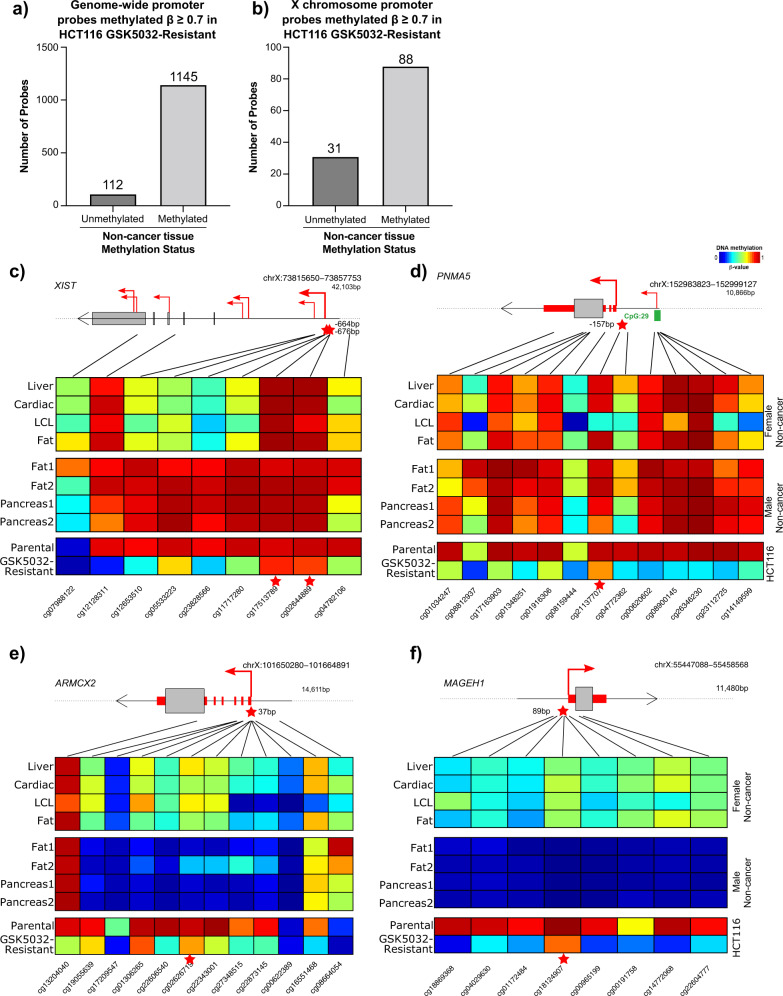
Hypermethylation is preferentially retained on the X-chromosome following long-term DNMT1 inhibition in HCT116 cells. **a** Number of retained methylation CpG promoter probes (*β*-value $\ge$ 0.7) following long-term DNMT1 inhibition in HCT116 cells (GSK5032-resistant) classified by the methylation status in non-cancer human tissues on all chromosomes (*n* = 1257 probes). **b** Number of retained methylation CpG promoter probes (*β*-value $\ge$ 0.7) following long-term DNMT1 inhibition in HCT116 cells (GSK5032-resistant) classified by the methylation status in non-cancer human tissues on the X chromosome (*n* = 119 probes). **c** Heatmap of DNA methylation levels for all EPIC probes associated with the *XIST* locus across a panel of non-cancer female tissues (top), non-cancer male tissues (middle), and HCT116 parental and long-term DNMT1 inhibition GSK5032-resistant cell lines (bottom). Red stars indicate specific promoter probes that were identified as retaining methylation following long-term DNMT1 inhibition in our analysis. Identified *XIST* promoter probes are an example of being methylated in non-cancer human tissues. **d** Example of a retained methylated promoter probe found in *PNMA5* that is methylated in non-cancer human tissues. **e** Example of a retained methylated promoter probe found in *ARMCX2* that is unmethylated in non-cancer human tissues. **f** Example of a retained methylated promoter probe found in *MAGEH1* that is unmethylated in non-cancer human tissues.

To simplify further investigation, we narrowed our analysis to the HCT116 cells before (parental) and after long-term DNMT1 inhibition (GSK5032-resistant) and selected probes resistant to demethylation (*β*-value ≥ 0.7) which were located in the vicinity of promoters. On a genome scale, we found that 112 of them (9%) had become *de novo* methylated in HCT116 parental cells (i.e., unmethylated in non-cancer tissues) whereas 1145 were already methylated in non-cancer uncultured human tissues (Fig. 3a). When we further narrowed our inquiry to the X chromosome, it was immediately apparent that a greater percentage (26%) of the resistant probes had acquired methylation *de novo* in HCT116 parental cells as these probes demonstrate typical methylation patterns of female X chromosome inactivation (unmethylated in males, monoallelic methylation (50%) in females) in non-cancer human tissue (Fig. 3b). Because the X chromosome probes are included in the analysis in Fig. 3a, the actual enrichment is 28% in that 31 of a total of 112 demethylation-resistant probes are located on the active X chromosome present in the HCT116 cell line. Moving forward, we continued to characterize the probes that retained DNA methylation in the HCT116 GSK5032-resistant cells based on their methylation status in non-cancer human tissues.

Next, we extended our analysis to examine the DNA methylation environment flanking the highly resistant CpG sites we had identified (Fig. 3c–f and Supplementary Fig. 4a). Two resistant probes located in the multiple transcription start sites (TSSs) of *XIST* were methylated in both female and male non-cancer tissues and in the HCT116 parental cells (Fig. 3c). Since the *XIST* gene is transcribed on the inactive but not the active X chromosome^15^, the functions of these CpGs, although highly resistant to demethylation, have unknown significance. On the other hand, the 4 probes downstream showed the expected behavior for an X-linked gene being monoallelically methylated in normal female cells and methylated completely in male and HCT116 parental cells. This region became less methylated after drug treatment, but the retained methylation may not be sufficient for silencing given that the treated cells do express *XIST* (see Table 1). Figure 3d shows regional analysis of a resistant probe in the region of the TSS of *PNMA5* demonstrating that retention of methylation was quite focal. The methylation patterns in non-cancer male and female cells were very similar suggesting that this gene is not subject to X inactivation.

**Table 1:** Transcripts per million (TPM) for genes with promoter probes that remain methylated (*β*-value ≥ 0.7) after longterm GSK5032 treatment in HCT116 cells that are methylated in cancer on the X chromosome.

Gene name	Non-cancerous colonic epithelium	HCT116 parental	HCT116 GSK5032-resistant	Gene description
PGK1	*89*	*74*	*114*	*Phosphoglycerate kinase 1*
HPRT1	*18*	*41*	*36*	*Hypoxanthine phosphoribosyltransferase 1*
**GPRASP1 (3)**	17	0	0	G protein-coupled receptor associated sorting protein 1
**MAGEH1 (1)**	22	0	1	MAGE family member H1
**MAGED1 (1)**	31	38	30	MAGE family member D1
**ARMCX2 (1)**	17	0	0	Armadillo repeat containing X-linked 2
**DKC1 (1)**	25	53	54	Dyskerin pseudouridine synthase 1
**PCSK1N (2)**	12	0	1	Proprotein convertase subtilisin/kexin type 1 inhibitor
**LRCH2 (2)**	6	0	0	Leucine rich repeats and calponin homology domain containing 2
**BEX1 (1)**	6	0	0	Brain expressed X-linked 1
**RAB9B (1)**	4	0	0	RAB9B, member RAS oncogene family
**ARMCX4 (1)**	5	0	0	Armadillo repeat containing X-linked 4
**GAB3 (1)**	4	0	1	GRB2 associated binding protein 3
BHLHB9 (2)	2	1	0	Basic helix-loop-helix family member b9
TMEM255A (2)	2	0	0	Transmembrane protein 255A
RPS6KA6 (1)	3	0	0	Ribosomal protein S6 kinase A6
FGF13 (1)	1	0	0	Fibroblast growth factor 13
DCAF12L2 (1)	0	0	0	DDB1 and CUL4 associated factor 12 like 2
GRIA3 (1)	1	0	0	Glutamate ionotropic receptor AMPA type subunit 3
COL4A6 (1)	1	1	3	Collagen type IV alpha 6 chain
NDP (1)	0	0	0	Norrin cystine knot growth factor NDP
KLHL34 (1)	0	0	0	Kelch like family member 34
INGX (1)	0	0	0	Inhibitor of growth family, X-linked (pseudogene)
GRP101 (1)	0	0	0	G protein-coupled receptor 101
ESX1 (1)	0	0	0	ESX homeobox 1
IRS4 (1)	0	0	0	Insulin receptor substrate 4
HTR2C (1)	0	0	0	5-hydroxytryptamine receptor 2C
DGKK (1)	0	0	0	Diacylglycerol kinase kappa
XIST (2)	0	2	94	X inactive specific transcript

Bold indicates genes expressed in non-cancerous transverse colonic epithelium.

Parentheses indicate the number of EPIC array promoter probes hypermethylated after long term DNMT1 inhibition.

Two examples of CpG probes resistant to drug induced demethylation in the HCT116 parental cells (*ARMCX2* and *MAGEH1*), showed the behavior expected for X-linked genes in non-cancer tissues (Fig. 3e, f). They, and other adjacent probes, were roughly 50% methylated in non-cancer female tissues and completely unmethylated in males. On the other hand, they were heavily methylated in the HCT116 parental line and maintained this DNA methylation after long-term DNMT1 inhibition (GSK5032-resistant). As the EPIC array probes provide a snapshot of the DNA methylation status of an individual CpG, we confirmed the EPIC array results by targeted strand specific bisulfite sequencing of the region surrounding the identified retained CpGs (Supplementary Fig. 4b, c). Importantly, targeted bisulfite sequencing of the region demonstrated that many CpGs flanking the EPIC probe retained DNA methylation following long-term DNMT1 inhibition (Supplementary Fig. 4b, c). Consistent with our EPIC array results, the specific CpGs corresponding to the probes in *XIST* and *ARMCX2* showed retention of methylation after drug treatment.

### Identified CpGs that retain hypermethylation in promoters on the X chromosome are frequently methylated in TCGA tumors

**Fig. 4:**
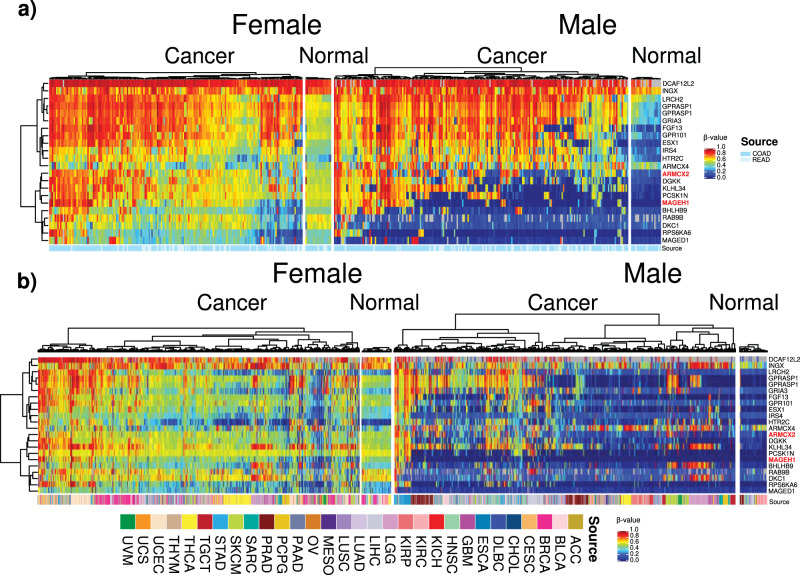
Hypermethylation of CpGs that are unmethylated in non-cancer human tissues is replicated in TCGA samples for colon and rectal adenocarcinoma as well as other cancer types. Heatmaps showing DNA methylation level as measured by the 22 HCT116 GSK5032-resistant probes (rows) in (**a**) COAD/READ samples (columns) (*n* = 402 COAD/READ tumor samples, *n* = 41 adjacent normal samples) (**b**) and a panel of cancer samples other than COAD/READ from TCGA data for identified probes that were unmethylated in non-cancer human tissues. A color spectrum of blue to red indicates low to high levels of DNA methylation (*β*-values ranging from 0 to 1). Samples were organized first by biological sex and then tissue type (cancer/normal). Genes discussed in the main text are labeled in red. Tumor type annotation is plotted beneath each heatmap, with color code explained as ‘source’ (*n* = 8790 human tumor samples, *n* = 714 adjacent normal samples).

Probes on the X chromosome are routinely excluded from methylation analysis in the TCGA and other data sets because their inclusion leads to strong clustering based on biological sex rather than on tumor type. We therefore queried TCGA data for the methylation status of CpG probes we had identified as unmethylated in non-cancer human tissues and as being highly resistant to DNA methylation inhibition in the cultured HCT116 parental and GSK5032-resistant cells. First, we examined data obtained using the now-obsolete Illumina 450 K arrays in colorectal and rectal tumors and adjacent normal tissue (Fig. 4a). We were able to do this for 22 probes which were shared on both arrays. With the exception of two probes (found in the promoters of genes *DFCAF12L2* and *INGX*), which were highly methylated in all samples, the remaining 20 probes showed the expected behavior in tumor-adjacent normal tissue in that they were substantially unmethylated in male tissue and about 50% methylated in females (similar pattern to the probes profiled in Fig. 3e, f). They also showed variable, but substantial *de novo* methylation in the tumors in both females and males (Fig. 4 and Supplementary Fig. 5a, b). This varied from almost universal methylation for the top 10 probes to more sporadic levels in the bottom 10. Indeed, evaluation of the distribution of β-values for individual probes of interest between tumor and adjacent normal tissues demonstrate significant hypermethylation of these CpG sites in COAD/READ tumors (Supplementary Fig. 5c). The *de novo* methylation of genes on the active X chromosome is therefore not sex-specific.

Interestingly, while the same probes showed similar behaviors in other normal tissues (except for *HTR2C* which appeared to be unmethylated in some female samples), there was clear evidence for *de novo* methylation across a panel of diverse cancer types (Fig. 4b). The methylation was not sex-specific but was markedly less evident than in the colorectal/rectal tumors displayed above (Fig. 4a). The methylation changes are therefore related to tumor type. We also queried the status of 62 of the probes categorized as being methylated in non-cancer and tumor tissues (Supplementary Fig. 6). Most of the probes behaved as expected in normal tissues although a minority were less methylated than anticipated particularly in colorectal tumors relative to the other cancer types examined. Our approach of using cultured colorectal tumor cells to isolate these probes therefore validates the value of concentrating on probes highly resistant to demethylation.

Finally, the expression levels of the genes corresponding to the methylated promoter probes were examined in normal colonic tissue reported in the GTEX database^16^ and compared to expression levels in the HCT116 parental and GSK5032-resistant cells (Table 1). Robust expression of the X-linked housekeeping genes, *PGK1* and *HPRT1* was seen in all samples as expected for these controls. Eleven genes (indicated by bold type) were expressed in normal colon, and nine of these were not expressed in HCT116 parental cells.

## Discussion

The recent development of a specific DNMT1 inhibitor, which does not require incorporation into DNA for its activity^17^, provided us with a useful tool to probe the role of the enzyme in the maintenance of DNA methylation patterns in cancer cell lines. Long term treatment of the HCT116 cells resulted in the derivation of cells which showed impaired growth rates and were markedly resistant to the compound. Importantly, we did not observe any mutations in the catalytic domain of DNMT1 that would account for the resistance observed and few changes were seen in the levels of mRNA for any of the DNMT isoforms examined; therefore, the mechanism of resistance remains unexplained.

Analysis of the patterns of residual methylation in the HCT116 and RKO GSK5032-resistant cells underscored the predominant role played by DNMT1 in maintaining DNA methylation in mammalian cells. Comparison of the patterns generated by inhibitor treatment with those in the HCT116 DKO1 line which has a complete knockout of *DNMT3B* and a hypomorphic version of *DNMT1* allowed us to gain further insight into the potential roles of the so-called *de novo* enzymes DNMT3A and DNMT3B in contributing to maintenance methylation. An interesting finding was that the HCT116 DKO1 line, which has no detectable DNMT3B, has less global and focal retained methylation than the resistant cells strongly suggesting a role for DNMT3B in maintenance of methylation in addition to its function in its establishment during development. The further delineation of these complementary roles of DNMT3B will require more experimentation than presented here.

The most surprising result of our analysis of the residual methylation patterns were the marked differences in the percentages of probes remaining methylated on individual chromosomes. The differences between the percentages of probes maintaining a *β*-value $\ge$ 0.7 varied by as much a 5 to 25 fold when comparing the lowest chromosome 9 to the highest chromosome X. The patterns of retained methylation on a per chromosome basis were remarkably similar at all β-value stringencies tested and in all cell lines. Since a common feature of all cell lines was the presence of DNMT3A, but not DNMT3B or a completely active DNMT1, a possible explanation of the result is that DNMT3A shows chromosome specificity in its ability to methylate DNA. Such specificity has not been noted before and it is not immediately apparent what might be driving such a pronounced variation between the macromolecular structures of chromosomes. Possible explanations include the existence of chromosome territories and differential nuclear organization^18^. Previously, Raddatz et al.^19^ showed that DNMT3A was able to maintain DNA methylation in large chromosome domains which had become hypomethylated in mouse tumors. Together with our data, it seems likely that DNMT3A shows methylation specificity over much larger domains than previously recognized.

It was remarkable that many of the same promoter CpGs found to be highly resistant to demethylation in culture were also *de novo* methylated in a considerable proportion of uncultured human colon and other tumors. Our expression analysis confirmed that many of the genes associated with these promoters were highly expressed in normal colonic tissue and silent in the HCT116 cell line (Table 1). While we have not formally established a link between the acquired methylation and gene silencing this seems to be quite likely. A possible explanation for the functions of these genes is that they are involved in cell viability and are selected for by the stringent requirement for continued growth in the presence of the inhibitor. Indeed, two of the examples we have highlighted, *ARMCX2* and *MAGEH1* (Fig. 3e, f), have previously demonstrated promoter hypermethylation and gene silencing in ovarian and liver cancer, respectively. Zeller et al identified *ARMCX2* as a primary target for acquired promoter DNA hypermethylation in cisplatin-resistant ovarian cancer cell lines, including cell lines derived from patients at relapse after becoming resistant to chemotherapy^20^. Wang et al. profiled the expression of MAGEH1 across liver cancer cell lines and hepatocellular carcinoma patients and determined that downregulation of MAGEH1 led to increased proliferation and poor prognosis^21^. Unfortunately, little is known about the functional roles of ARMCX2 and MAGEH1 aside from their expression patterns in non-cancer human tissues^22,23^. Taken together with our results, our approach has identified potential tumor-suppressor genes that warrant further investigation for their functional roles’ in both non-cancerous and cancerous human cells.

Whatever the mechanism, the retention of DNA methylation in these promoters and their potential functions in cancer have largely been overlooked given the difficulties of deciphering DNA methylation patterns and gene expression changes on X-linked genes. We are therefore reanalyzing databases for their possible functions in cancer cells.

## Methods

### Cell culture

HCT116 parental and RKO parental cells were obtained fresh from ATCC (ATCC® CCL-247™, ATCC® CRL-2577™; Manassas, VA) and cultured in McCoy’s 5A (Life Technologies, 16600–082; Waltham MA) supplemented with 10% heat-inactivated fetal bovine serum, 1% penicillin/streptomycin, and RPMI (Gibco, 11875–093; Gaithersburg, MD) supplemented with MEM Non-Essential Amino Acids (Life Technologies, 11140–050; Waltham MA), sodium pyruvate (Life Technologies, 11360-070; Waltham MA), 10% heat-inactivated fetal bovine serum, 1% penicillin/streptomycin, respectively. Cells were treated with increasing doses of GSK5032 (GlaxoSmithKline; Collegeville, PA) starting with 200 nM and ultimately reaching 7400 nM over the course of 150 days. Fresh media and inhibitor were given to the cells every 3–4 days, passing the cells as needed. All cell lines tested negative for Mycoplasma contamination throughout the duration of the experiments. HCT116 and RKO GSK5032-resistant cell lines are available upon request. HCT116 DKO1 cells were kindly provided by Dr. Stephen B. Baylin and cultured in McCoy’s 5A (Life Technologies, 16600-082; Waltham MA) supplemented with 10% heat-inactivated fetal bovine serum and 1% penicillin/streptomycin.

### Cell viability

IC50 curves were generated using FluoReporter™ Blue Fluorometric dsDNA Quantitation Kit (Invitrogen, F2962; Waltham MA) according to manufacturer’s instructions. Briefly, cells were plated in 96 well white walled plates (Corning, 3601; Corning, NY) and treated with 13 increasing two-fold dilutions of GSK5032. Plates were harvested on day 5 after treatment and processed. Fluorescence was measured with excitation/emission wavelengths were measured at 360 nm/460 nm using a Synergy HT multimode microplate reader (BioTek; Winooski, VT).

### RNA isolation, cDNA synthesis, qPCR

Cells were harvested for RNA by adding TRIzol (ThermoFisher, 15596026; Waltham, MA) directly to the plate to lyse the cells. The Direct-zol™ RNA MiniPrep Plus kit (Zymo Research, R2072; Irvine, CA) with DNaseI digestion was used to extract RNA. RNA concentration was determined with a Nanodrop Lite (ThermoScientific; Waltham, MA) then samples were normalized to the same concentration. The High-Capacity cDNA Reverse Transcription Kit (ThermoFisher, 4368814; Waltham, MA) was used to generate cDNA with 2ug input RNA according to the manufacturer’s instructions.

After cDNA was diluted 4 fold, 4 μL of cDNA was used in a 20 μL qPCR reaction with 250 nM primers and 10 μL KAPA SYBR® FAST qPCR Master Mix (2X) (Kapa Biosystems, KK4618; Wilmington, MA). Primers (Supplementary Table 1) were used to measure gene expression. Displayed values are ΔΔCq values normalized to TBP expression. Each graph represents the average of three replicates with standard error plotted.

### DNA isolation and EPIC array

DNA was isolated using the DNeasy Blood and Tissue kit (Qiagen, 69504; Germantown, MD) according to manufacturer’s instructions, then submitted to the Van Andel Institute Genomics Core for processing on Illumina’s Infinium MethylationEPIC BeadChip.

### EPIC array processing

EPIC array image data (idat) files were read into R (Version > 3.6) and processed using the SeSAMe package (version 1.8.2) using the standard settings^24^. EPIC array probes with a detection *p*-value > 0.05 were excluded from the analysis as probes that fail this check are unable to provide reliable *β*-value calculations.

### Enrichment bias calculation and hypergeometic distribution testing

Enrichment bias calculations were done by first determining the following values for each chromosome:

*q* = Number of CpGs that retained DNA methylation in the DNMT compromised cell lines (DKO1; GSK5032-resistant) (*β*-value $\ge$ 0.7)

*m* = Total number of CpGs on the EPIC array with high DNA methylation (*β*-value $\ge$ 0.7) in the parental cells (HCT116; RKO)

*n* = Total number CpGs on the EPIC array that do not match feature

*k* = Total number of all CpGs with DNA methylation (*β*-value $\ge$ 0.7) in the DNMT compromised cell lines (DKO1; GSK5032-resistant).

Next, the expected number of CpGs that would retain DNA methylation on each chromosome by random chance was determined with the following equation:

1 $$e=\,\left(\frac{m}{m+n}\right)k$$

Finally, percent enrichment bias was calculated with the following equation:

2 $$\% \,{{{{{\rm{enrichment}}}}}}\,{{{{{\rm{bias}}}}}}=\,\left(\frac{q-e}{k}\right)\times 100$$

Where positive or negative enrichment values indicate more or less enrichment for a chromosome than would be expected by random chance, respectively.

Hypergeometric distribution testing for determining significance of enrichment bias was performed using the phyper() function in R (Version > 3.6) with the following values: *q, m, n, k*.

### Categorization of probe methylation status in non-cancer human tissues

Number of probes remaining methylated after long term, high dose GSK5032 treatment were determined using a *β*-value cut off of $\ge$ 0.7. Using a panel of non-cancerous male and female EPIC datasets (Supplementary Table 2), probes were called to be unmethylated when they were unmethylated in males or 50% methylated in females for probes on the X chromosome, but were greater than 70% methylated in HCT116 parental and GSK5032-resistant cells. Probes were considered to be methylated when they also had a high beta value (*β*-value $\ge$ 0.7) in the non-cancerous male and female tissues.

### TCGA analysis

DNA methylation array data (Infinium HumanMethylation450 BeadChip; HM450) and clinical data were downloaded from the GDC Data Portal (https://portal.gdc.cancer.gov/repository), which includes 8790 and 714 whitelisted human tumor and adjacent normal samples, respectively, across 32 different tumor types. Among the 119 EPIC array probes, 84 are present on the HM450 array. Heatmaps were generated in RStudio (Version > 3.6), with samples split based on clinical biological sex along with subdivisions into either COAD/READ or non-COAD/READ tumor and their adjacent normal.

### TCGA statistical analysis

To test if the amount of methylation across all 22 CpGs of interest differed between the COAD/READ and normal tissues, a beta mixed-effects model with random intercepts for both CpG and patient ID was fit using R (Version > 3.6) via the glmmTMB package (https://cran.r-project.org/web/packages/glmmTMB/index.html). An interaction between biological sex and group were included to allow the mean methylation to vary by biological sex (which was expected due to the number of X chromosomes present in biological males versus females). To investigate differential methylation between COAD/READ samples and normal tissue at a subset of specific loci (cg02626719, cg18124907, cg19355555, cg24401557, and cg12950441) beta mixed-effects regressions with random intercepts for each patient and an interaction for biological sex were used. cg18124907 was an exception due to poor diagnostics assuming a beta distribution and was instead analyzed using a semi-parametric ordinal mixed-effects model (https://cran.microsoft.com/snapshot/2017-08-01/web/packages/ordinal/index.html).

### Statistics and reproducibility

Statistical analyses were performed as described in the methods section and figure legends. In all cases, an independent biological replicate indicates that cells were plated and treated with drugs completely separately and on completely different days for a true biological replicate. For Fig. 1a, the biological replicates for the IC50 curves were 5 for HCT116 Parental and 7 for HCT116 GSK5032-resistant. The biological replicates for RKO Parental and RKO GSK5032-resistant were 3 each. The EPIC data was generated from one biological replicate for each cell line and contained a total of 627,029 probes that were included in the analysis for the rest of the figures.

For Supplementary Fig. 1a, the cell counts had the biological replicates as indicated by the number in parentheses for HCT116 Parental (9), HCT116 GSK5032-resistant (6), HCT116 DKO1 (4), RKO Parental (5), and RKO GSK5032-resistant (4). For Supplementary Fig. 1b, the qPCR data and standard error were calculated from three biological replicates for each sample. Biological replicates were from separate preparations of RNA that was extracted and converted to cDNA independently as well.

### Reporting summary

Further information on research design is available in the Nature Research Reporting Summary linked to this article.

## Supplementary information

Supplementary Information

Description of Additional Supplementary Files

Supplementary Data 1

Reporting Summary

## Data Availability

All Illumina Infinium MethylationEPIC array data is deposited in GEO and available under accession GSE182209. Source data for figures are available in Supplementary Data 1.
