# Supplementary material for: Chromosome-specific retention of cancer-associated DNA hypermethylation following pharmacological inhibition of DNMT1
Source: Commun Biol. 2022 Jun 2;5:528. doi: 10.1038/s42003-022-03509-3 (PMC9163065; doi:10.1038/s42003-022-03509-3)
Supplement: Supplementary file 1 — Supplementary Information [file 42003_2022_3509_MOESM1_ESM.pdf]

Supplemental Information

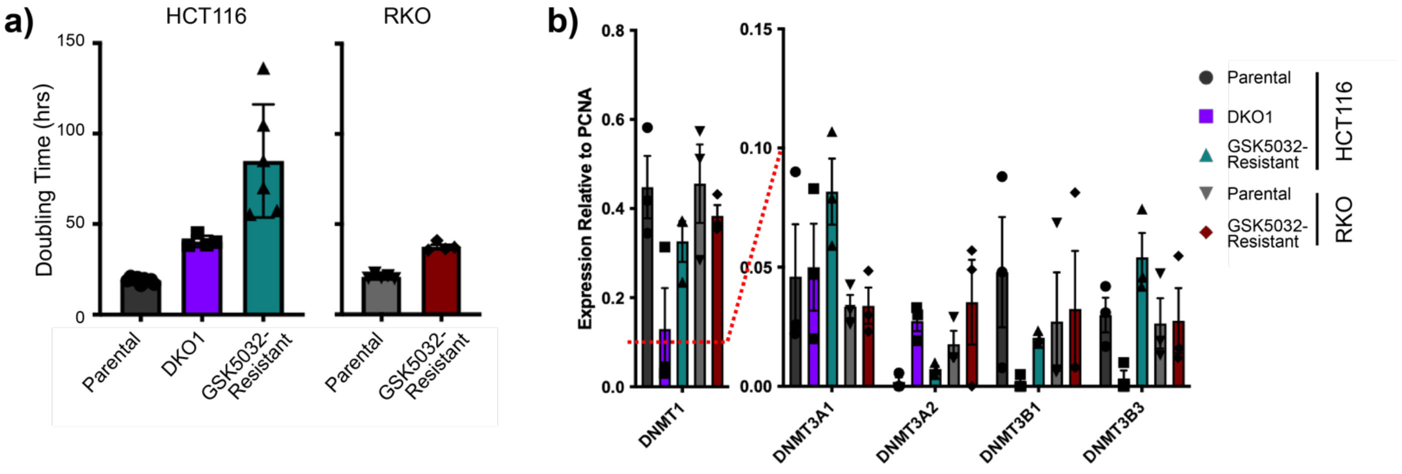

Supplementary Figure 1. HCT116 and RKO GSK5032-resistant cells demonstrate slowed cell proliferation, but unaltered DNMT isoform gene expression. a) Average doubling time of indicated cell lines in culture. Cells were counted after resistance was reached, and average doubling time of cell culture populations were calculated based on the number of cells plated, the number of cells at the time of passing, and the amount of time in hours that had passed. Error bars represent the standard error (n = 11 for HCT116 Parental, n= 3 for HCT116 DKO1, n = 13 for HCT116 GSK5032-Resistant, n = 5 for RKO Parental, n = 4 for RKO GSK5032-Resistant). **b)** Quantitative DNMT isoform expression analysis (qRT-PCR) among all cell lines. DNMT isoform expression is normalized to *PCNA* expression under each condition. Error bars represent the standard error (n = 3).

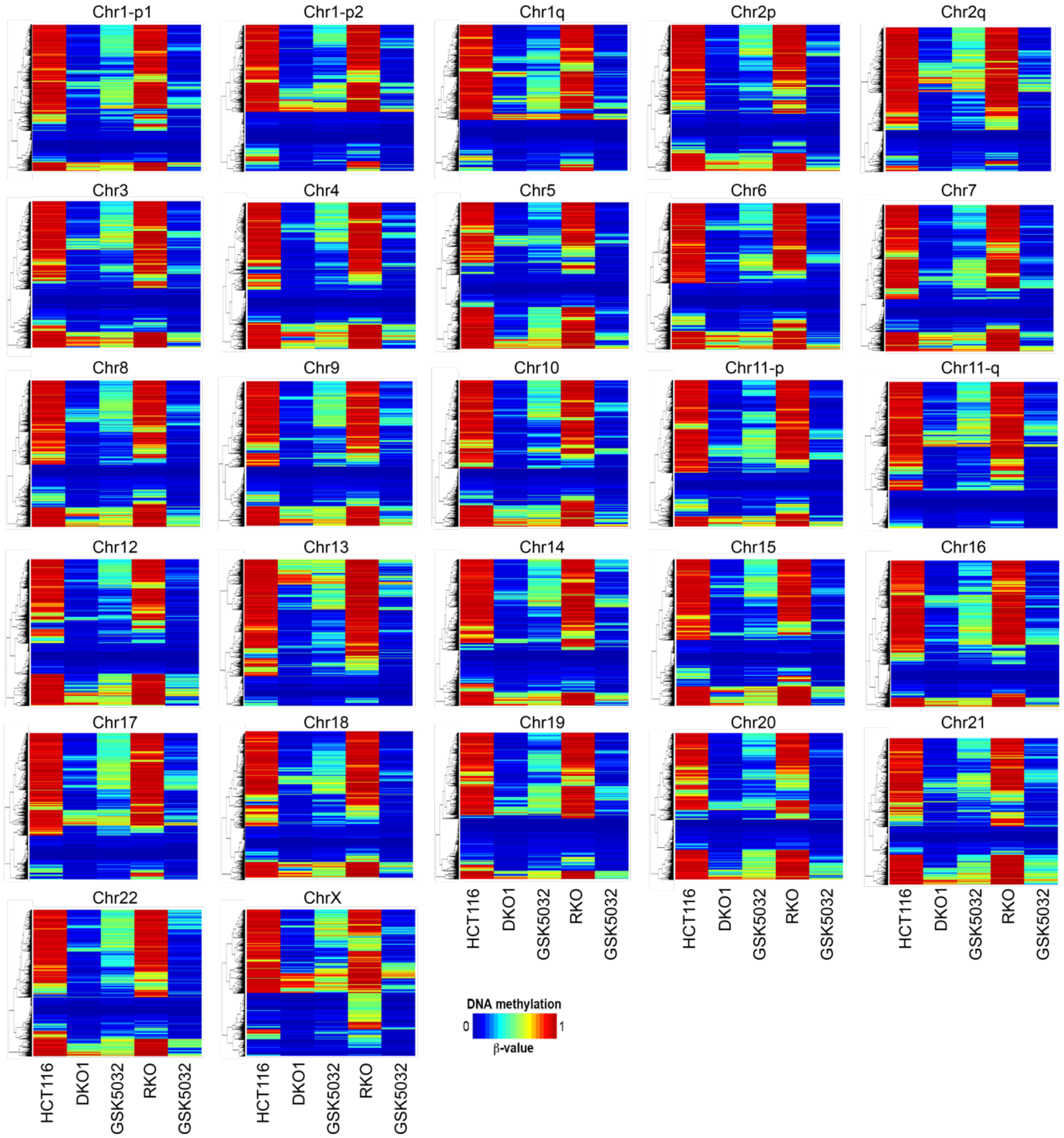

Supplementary Figure 2. CpG probes remaining methylated in HCT116 and RKO after DNMT1 inhibition (GSK5032-resistant) are enriched on the X chromosome. Heatmaps of DNA methylation level (β-value) of all the CpG probes present on the indicated chromosome. Chromosomes 1, 2, and 11 were subdivided into chromosomal arms due to the large number of probe representation and computational limitations of generating the heatmaps. β-values are represented by color scale where dark blue (colder colors) indicates no methylation and red (warmer colors) indicates complete methylation. Chr1 (n = 60782), Chr2 (n = 45297), Chr3 (n = 33938), Chr4 (n = 23258),

Chr5 (n = 30710), Chr6 (n = 36837), Chr7 (n = 31375), Chr8 (n = 27924), Chr9 (n = 19830), Chr10 (n=30572), Chr11 (n = 37567), Chr12 (n = 32439), Chr13 (n = 13597), Chr14 (n = 21490), Chr15 (n = 21016), Chr16 (n = 29956), Chr17 (n = 36207), Chr18 (n = 10513), Chr19 (n = 31315), Chr20 (n = 18727), Chr21 (n = 7067), Chr22 (n= 14929), ChrX (n = 10678)

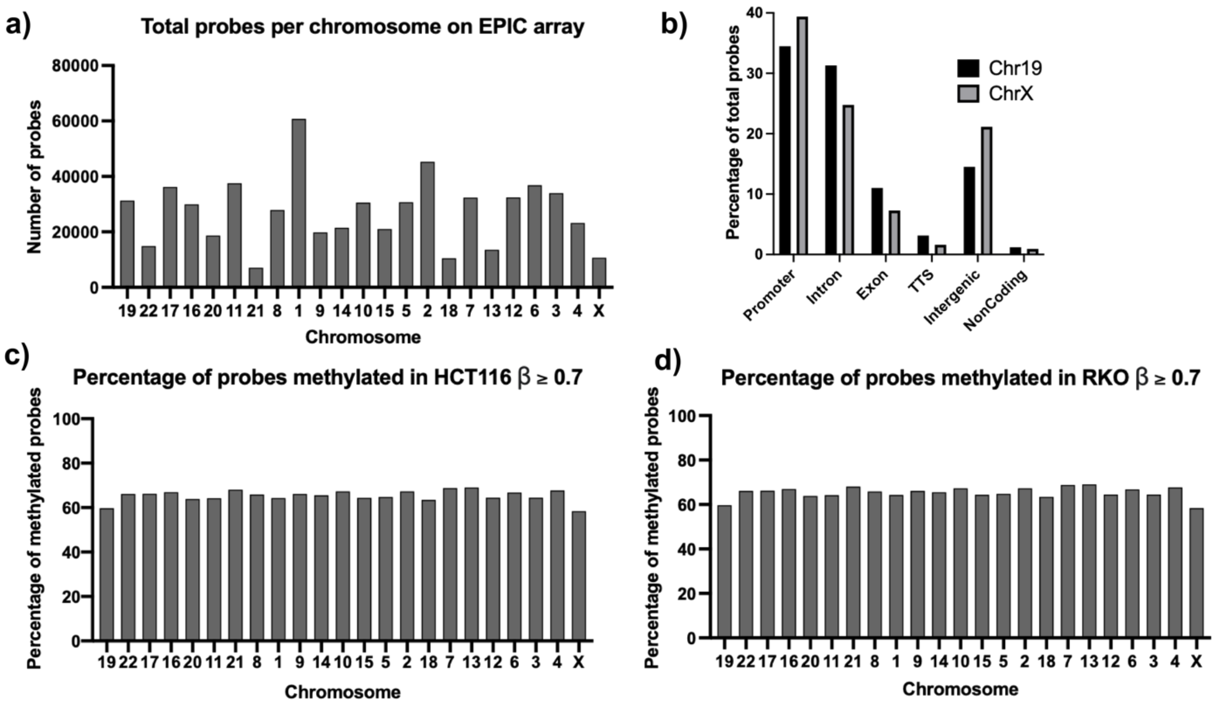

Supplementary Figure 3. The enrichment of methylated probes on the X chromosome is not dependent on the distribution of the probes on the EPIC array nor on the methylation state of probes prior to treatment. a) Number of EPIC array probes per chromosome. Chromosomes are ordered based on the percentage of methylated probes in HCT116 GSK5032-resistant cells as shown in Figure 2b. **b)** Genomic annotation for methylated probes after GSK5032 treatment for the least enriched (19) and the most enriched (X) chromosomes **c)** Percentage of EPIC array probes on each chromosome that are methylated in HCT116 parental cells (β-value ≥ 0.7). **d)** Percentage of EPIC array probes on each chromosome that are methylated in RKO parental cells (β-value ≥ 0.7).

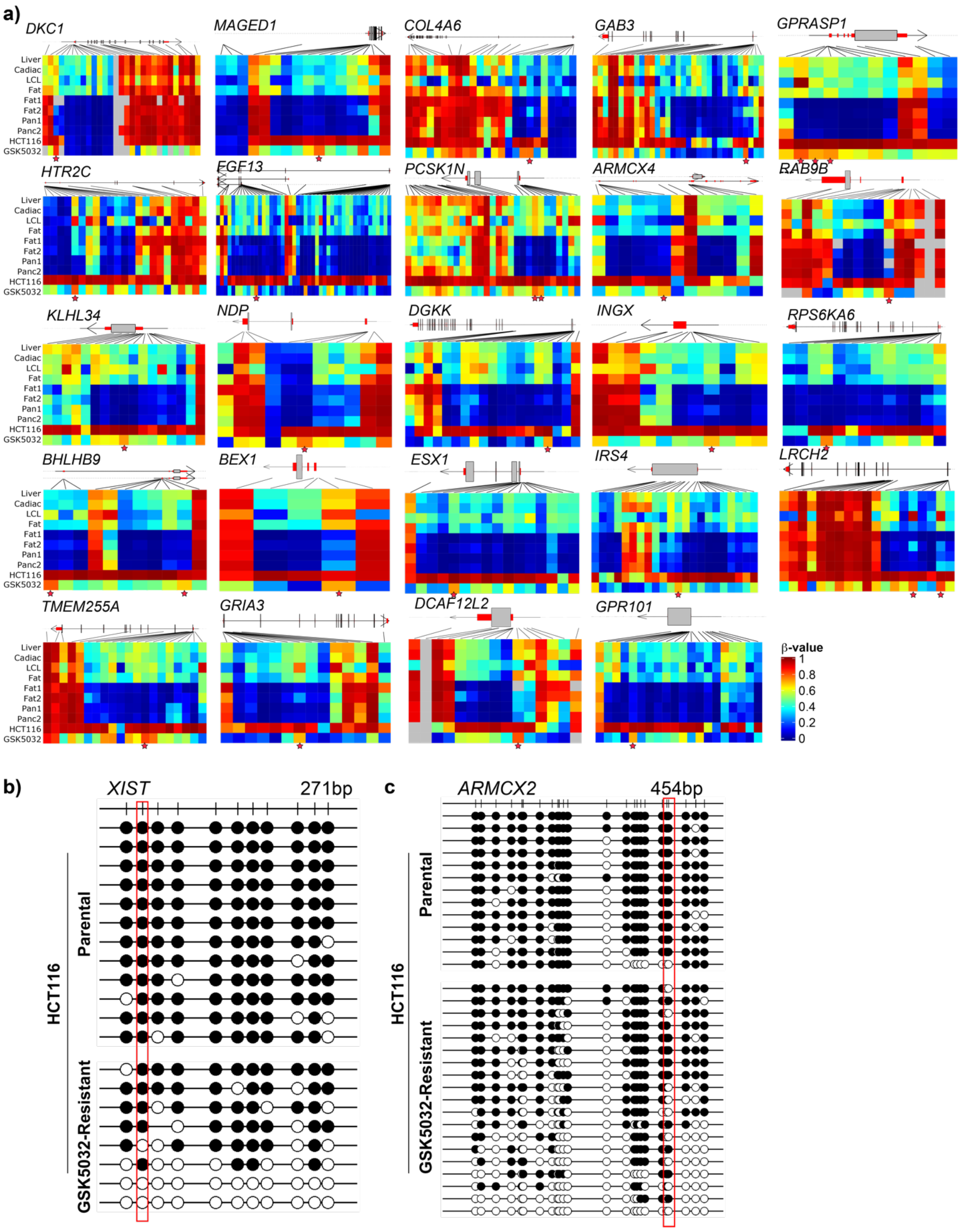

Supplementary Figure 4. Retained hypermethylation of probes unmethylated in non-cancer tissue in the promoters of genes located on the X chromosome. a)

Heatmaps of all X chromosome genes that were identified as retaining methylation following long-term DNMT1 inhibition in HCT116 cells (GSK5032-resistant) at CpGs that are unmethylated in non-cancer human tissue. Red stars indicate specific promoter probes that were identified as retaining methylation following long-term DNMT1 inhibition in our analysis. Order of non-cancer tissues is same as described in Figure 3c-f. **b)** Targeted bisulfite sequencing for region around methylated probe near *XIST* (red box) that was identified in 3c. **c)** Targeted bisulfite sequencing for region around methylated probe near *ARMCX2* (red box) that was identified in 3e.

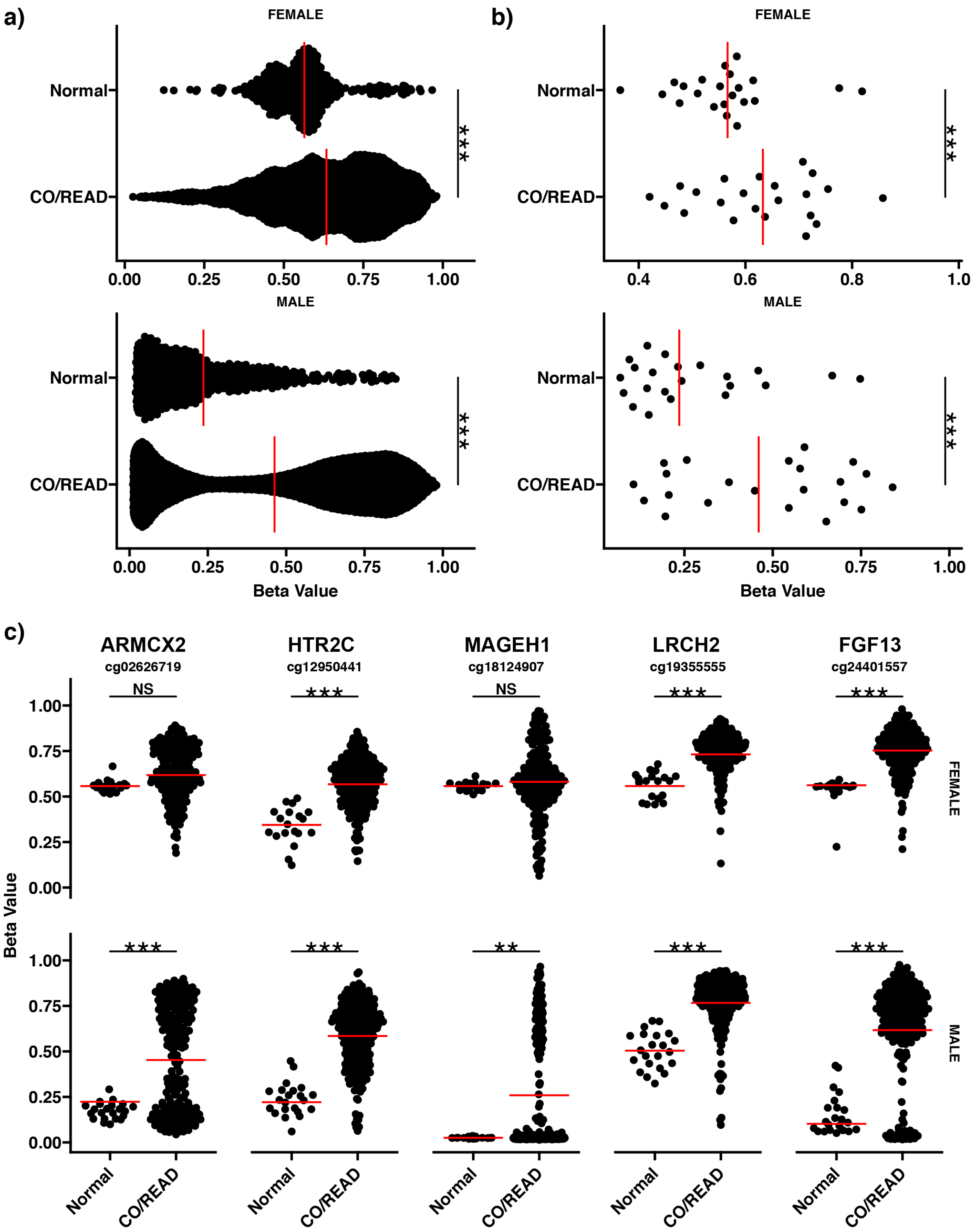

a)
FEMALE
Normal
CO/READ
***
0.00
0.25
0.50
0.75
1.00
MALE
Beta Value
b)
0.4
0.6
0.8
1.0
c)
ARMCX2
cg02626719
HTR2C
cg12950441
MAGEH1
cg18124907
LRCH2
cg19355555
FGF13
cg24401557
NS
**
Beta Value

Supplementary Figure 5. DNA methylation status of COAD/READ patient data for identified CpG probes that are unmethylated in non-cancer tissues. a) β-value distribution of all CpG probes represented in Figure 4a categorized by COAD/READ and Normal patient data, stratified by biological sex. **b)** Average β-value of each CpG probe represented in Figure 4a across Normal tissue and COAD/READ, stratified by biological sex. Each dot represents the average β-value across TCGA patient data of an individual CpG that was identified from the status of DNA methylation in non-cancer tissue analysis. Averages were estimated via beta mixed-effects model with random intercepts for each patient and a three way interaction between group, biological sex, and CpG. The mean of the averages was also estimated from this model, pooled across all CpGs. **c)** β-value distribution of the specified CpG probe (*ARMCX2*, *HTR2C*, *MAGEH1*, *LRCH2*, *FGF13*) across Normal and Cancer (COAD/READ) tissue, stratified by biological sex.

Red lines represent the expected mean β-value for each biological sex and were estimated from a respective beta mixed-effects model with the exception of *MAGEH1* which was estimated using an ordinal mixed-effects regression. '**' $p < 0.01$, '***' $p < 0.001$, 'NS' $p > 0.05$. Female Normal (n = 19); Female CO/READ (n = 186); Male Normal (n = 22); Male CO/READ (n = 216).

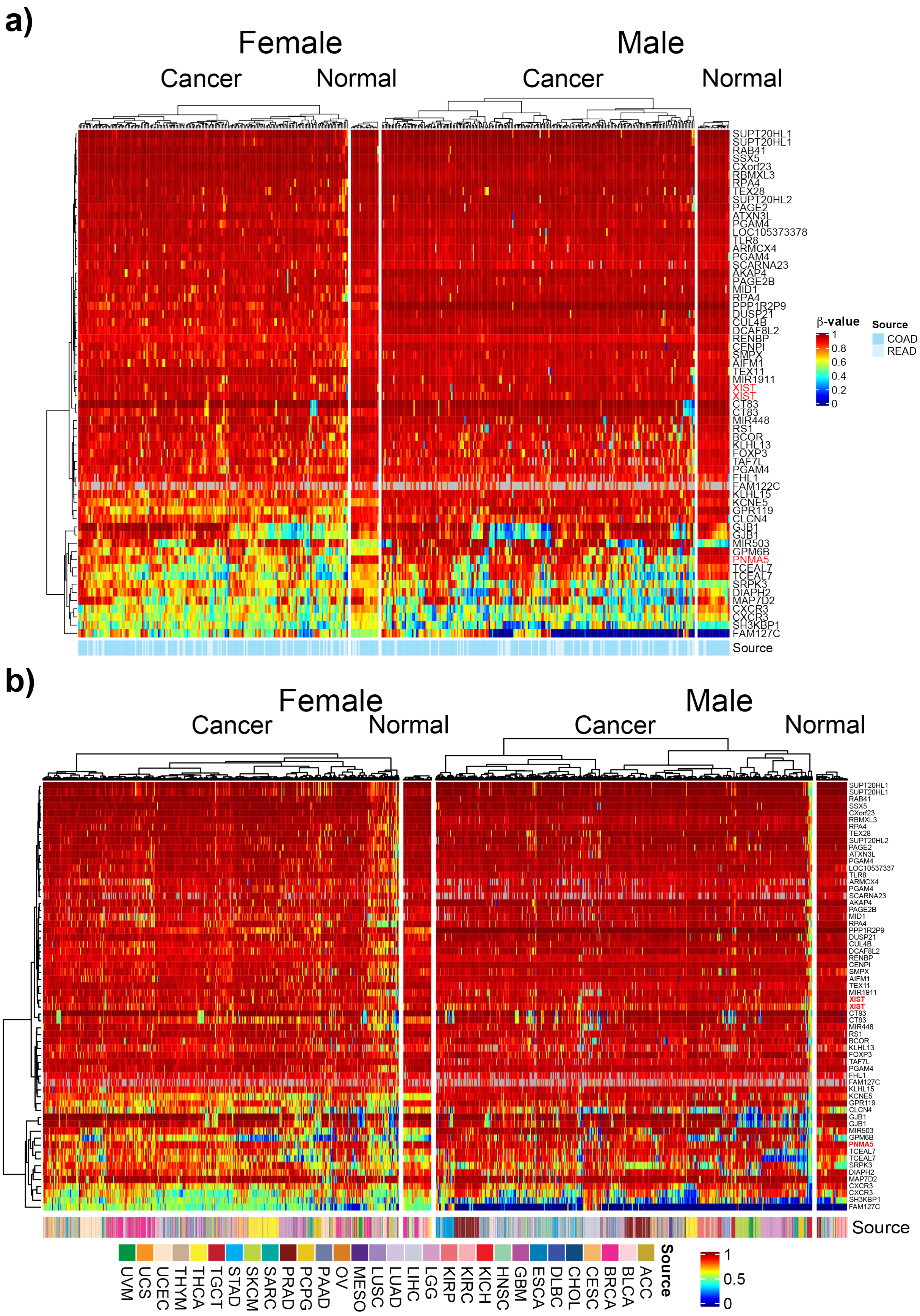
a)
Female
Male
Cancer
Normal
Cancer
Normal
SUPT20HL1
SUPT20HL1
RAB41
SSX5
CXorf23
RBMXL3
RPA4
TEX28
SUPT20HL2
PAGE2
ATXN3L
PGAM4
LOC105373378
TLR8
ARMCX4
PGAM4
SCARNA23
AKAP4
PAGE2B
MID1
RPA4
PPP1R2P9
DUSP21
CUL4B
DCAF8L2
RENBP
CENPI
SMPX
AIFM1
TEX11
MIR1911
XIST
XIST
CT83
CT83
MIR448
RS1
BCOR
KLHL13
FOXP3
TAF7L
PGAM4
FHL1
FAM122C
KLHL15
KCNE5
GPR119
CLCN4
GJB1
GJB1
MIR503
GPM6B
PNMA5
TCEAL7
TCEAL7
SRPK3
DIAPH2
MAP7D2
CXCR3
CXCR3
SH3KBP1
FAM127C
Source
β-value
1
0.8
0.6
0.4
0.2
0
Source
COAD
READ
b)
Female
Male
Cancer
Normal
Cancer
Normal
Source
UVM
UCS
UCEC
THYM
THCA
TGCT
STAD
SKCM
SARC
PRAD
PCPG
PAAD
OV
MESO
LUSC
LUAD
LIHC
LGG
KIRP
KIRC
KICH
HNSC
GBM
ESCA
DLBC
CHOL
CESC
BRCA
BLCA
ACC
Source
1
0.5
0

Supplementary Figure 6. DNA methylation patterns for probes identified as methylated in non-cancer tissues in TCGA samples for colon and rectal adenocarcinoma as well as other cancers. DNA methylation heatmaps for **a)** COAD/READ samples (CO/READ (n = 402); Normal (n = 41)) and **b)** a panel of cancer samples other than COAD/READ from TCGA data for probes identified as methylated in non-cancer tissue. Samples (as columns) are organized first by biological sex and then tissue type (cancer/normal). Probes are plotted as rows, with DNA methylation levels indicated by a spectrum of blue to red for low (0%) to high (100%) DNA methylation. Genes discussed in the main text are labeled in red. Tumor type annotation is plotted beneath each heatmap, with color code explained as 'source' (n = 8,790 human tumor samples, n = 714 adjacent normal samples).

Supplementary Table 1: Primers for qPCR

Gene	Forward Primer	Reverse Primer
DNMT1	5'-GGTTCTTCCTCCTGGAGAATGTC-3'	5'-GGGCCACGCCGTACTG-3'
DNMT3A1	5'- GGGGGACCCCTACTACATCA-3'	5'-CACAGCATTCATTCCTGCAA-3'
DNMT3A2	5'-CGAGTTCTGGAGATGCTGACT-3'	5'-ACCTTGGCTTTCTTCTCAGC-3'
DNMT3B1	5'-CATCTCACGGTTCCTGGAGTGTA-3'	5'-TCCTGCAGCTCGAGTTTATCA-3'
DNMT3B3	5'-TACCCGGGATGAACAGGATCT-3'	5'-AGTAGTCCTTCAGAGGGGCG-3'
PCNA	5'-GCAGATGTACCCCTTGTTGTAGAGT-3'	5'-TCTTCATCCTCGATCTTGGGA-3'

Supplementary Table 2: ENCODE Accession IDs for DNA methylation datasets used in the study

**Sample Name in Manuscript**	**ENCODE Accession ID**
Liver (female)	ENCSR937LY2
Cardiac (female)	ENCSR517JQA
GM12878, LCL (female)	ENCSR000ACX
Fat (female)	ENCSR306JCS
Fat1 (male)	ENCSR662NBA
Fat 2 (male)	ENCSR733WXF
Pancreas 1 (male)	ENCSR705PPD
Pancreas 2 (male)	ENCSR922EBK
