# Supplementary material for: Chromosome-specific retention of cancer-associated DNA hypermethylation following pharmacological inhibition of DNMT1
Source: Commun Biol. 2022 Jun 2;5:528. doi: 10.1038/s42003-022-03509-3 (PMC9163065; doi:10.1038/s42003-022-03509-3)
Supplement: Supplementary file 2 — Description of Additional Supplementary Files [file 42003_2022_3509_MOESM2_ESM.pdf]

Description of Additional Supplementary Files

File name: Supplementary Data 1
Description: The source data behind the graphs in the paper (Figure 1a, Figure 2b, Figure 2c, Figure 3a, Figure 3b). Each page of the excel file is labeled for the figure for which the data represents.
