# Supplementary material for: Chromosome-specific retention of cancer-associated DNA hypermethylation following pharmacological inhibition of DNMT1
Source: Commun Biol. 2022 Jun 2;5:528. doi: 10.1038/s42003-022-03509-3 (PMC9163065; doi:10.1038/s42003-022-03509-3)
Supplement: Supplementary file 4 — Reporting Summary [file 42003_2022_3509_MOESM4_ESM.pdf]

nature research

Corresponding author(s): Peter A. Jones
Last updated by author(s): 05/13/2022

Reporting Summary

Nature Research wishes to improve the reproducibility of the work that we publish. This form provides structure for consistency and transparency in reporting. For further information on Nature Research policies, see our Editorial Policies and the Editorial Policy Checklist.

Statistics

For all statistical analyses, confirm that the following items are present in the figure legend, table legend, main text, or Methods section.

n/a	Confirmed	
☐	☒	The exact sample size (n) for each experimental group/condition, given as a discrete number and unit of measurement
☐	☒	A statement on whether measurements were taken from distinct samples or whether the same sample was measured repeatedly
☐	☒	The statistical test(s) used AND whether they are one- or two-sided<br>*Only common tests should be described solely by name; describe more complex techniques in the Methods section.*
☐	☒	A description of all covariates tested
☐	☒	A description of any assumptions or corrections, such as tests of normality and adjustment for multiple comparisons
☐	☒	A full description of the statistical parameters including central tendency (e.g. means) or other basic estimates (e.g. regression coefficient) AND variation (e.g. standard deviation) or associated estimates of uncertainty (e.g. confidence intervals)
☒	☐	For null hypothesis testing, the test statistic (e.g. F, t, r) with confidence intervals, effect sizes, degrees of freedom and P value noted<br>*Give P values as exact values whenever suitable.*
☒	☐	For Bayesian analysis, information on the choice of priors and Markov chain Monte Carlo settings
☒	☐	For hierarchical and complex designs, identification of the appropriate level for tests and full reporting of outcomes
☒	☐	Estimates of effect sizes (e.g. Cohen's d, Pearson's r), indicating how they were calculated

Our web collection on statistics for biologists contains articles on many of the points above.

Software and code

Policy information about availability of computer code

Data collection	Illumina Infinium MethylationEPIC BeadChips were scanned and processed on the Illumina iScan system.
Data analysis	RStudio (R Version > 3.6); SeSAMe (version 1.8.2)

For manuscripts utilizing custom algorithms or software that are central to the research but not yet described in published literature, software must be made available to editors and reviewers. We strongly encourage code deposition in a community repository (e.g. GitHub). See the Nature Research guidelines for submitting code & software for further information.

Data

Policy information about availability of data

All manuscripts must include a data availability statement. This statement should provide the following information, where applicable:

- Accession codes, unique identifiers, or web links for publicly available datasets
- A list of figures that have associated raw data
- A description of any restrictions on data availability

All Illumina Infinium MethylationEPIC array data is deposited in GEO under accession GSE182209. ENCODE Accession IDs for DNA Methylation datasets: ENCSR937LY2 (liver, female), ENCSR517JQA (cardiac, female), ENCSR000ACX (GM12878 LCL, female), ENCSR306JCS (fat, female), ENCSR662NBA (fat1, male), ENCSR733WXF (fat2, male), ENCSR705PPD (pancreas 1, male), ENCSR922EBK (pancreas 2, male). TCGA DNA methylation array data and clinical data were downloaded from the GDC Data Portal (https://portal.gdc.cancer.gov/repository).

Field-specific reporting

Please select the one below that is the best fit for your research. If you are not sure, read the appropriate sections before making your selection.

☒ Life sciences ☐ Behavioural & social sciences ☐ Ecological, evolutionary & environmental sciences

For a reference copy of the document with all sections, see nature.com/documents/nr-reporting-summary-flat.pdf

Life sciences study design

All studies must disclose on these points even when the disclosure is negative.

Sample size	No statistical methods were applied to determine sample size. HCT116 and RKO GSK5032-resistant cell lines were derived once, and genomic DNA was isolated from all cell lines once for application to the EPIC array. For all assays characterizing the doubling-time and expression of DNMT isoforms across the cell culture models, experiments were conducted with at least 3 biological replicates.
Data exclusions	EPIC array probes with a detection pval > 0.05 were excluded from the analysis as probes that fail this check are unable to provide reliable beta-value calculations.
Replication	Results were replicated across three different models of DNMT1-depletion (HCT116 GSK5032-resistant; HCT116 DKO1; RKO GSK5032-resistant). We additionally performed locus specific bisulfite sequencing of candidate loci to verify the results from the EPIC array. All other experimental assays were successfully replicated across a minimum of 3 biological replicates.
Randomization	No randomization was conducted as it is not relevant to the current study.
Blinding	No blinding was conducted as it is not relevant to the current study.

Reporting for specific materials, systems and methods

We require information from authors about some types of materials, experimental systems and methods used in many studies. Here, indicate whether each material, system or method listed is relevant to your study. If you are not sure if a list item applies to your research, read the appropriate section before selecting a response.

Materials & experimental systems

n/a	Involved in the study	
☒	☐	Antibodies
☐	☒	Eukaryotic cell lines
☒	☐	Palaeontology and archaeology
☒	☐	Animals and other organisms
☒	☐	Human research participants
☒	☐	Clinical data
☒	☐	Dual use research of concern

Methods

n/a	Involved in the study	
☒	☐	ChIP-seq
☒	☐	Flow cytometry
☒	☐	MRI-based neuroimaging

Eukaryotic cell lines

Policy information about cell lines

Cell line source(s)	HCT116 parental and RKO parental lines were purchased fresh from ATCC. HCT116 DKO1 cell line was kindly provided by Dr. Stephen Baylin.
Authentication	Purchased cell lines were not authenticated as they were freshly supplied from ATCC. HCT116 DKO1 cell line was authenticated by quantitative reverse-transcriptase PCR of DNMT1 and DNMT3B mRNA.
Mycoplasma contamination	All cell lines tested negative for Mycoplasma contamination.
Commonly misidentified lines (See ICLAC register)	No commonly misidentified cell lines were used in the study.
